# Unilateral buphthalmos, corneal staphyloma and corneal fistula caused by pathogenic variant in the PITX3 gene: a case report

**DOI:** 10.1186/s12886-022-02573-x

**Published:** 2022-09-24

**Authors:** Lin Zhou, Zhike Xu, Qianying Wu, Xin Wei

**Affiliations:** 1 Department of ophthalmology, West China Hospital, Sichuan University, Address 37, Guo Xue Lane, Chengdu, 610041 Sichuan China; 2 Department of ophthalmology, The people’s hospital of Leshan, Leshan, 614700 China

**Keywords:** *PITX3*, Variant, Unilateral buphthalmos, Corneal staphyloma, Corneal fistula

## Abstract

**Introduction:**

*PITX3* has been reported to be associated with congenital cataracts, anterior segment mesenchymal dysgenesis, Peters’ anomaly, and microphthalmia. In this case, an infant with unilateral buphthalmos, corneal staphyloma and corneal fistula carrying a variant in PITX3 was reported.

**Case description:**

We describe a 4-month-old female infant who was referred to our Eye Clinic because of gradual enlargement of the eyeball in the right eye and whitish opacity in both eyes. Buphthalmos with long axial length (22.04 mm), macrocornea with diffuse corneal oedema and opacity (14.50 mm*14.50 mm) and high intraocular pressure (23.78 mmHg) were detected in the right eye. Microphthalmia with short axial length (16.23 mm), microcornea with diffuse corneal oedema and opacity (7.50 mm*6.50 mm) were detected in the left eye. A 360° trabeculotomy was performed for the right eye. However, corneal staphyloma and corneal fistula in the right eye were detected 6 months after the surgery. A variant in exon 4 of *PITX3* (c.640_656dup (p. Gly220Profs*95)) was identified in the proband but was not detected in her healthy parents.

**Conclusion:**

A novel phenotype characterized by unilateral buphthalmos, corneal staphyloma and corneal fistula in an infant were reported to be associated with *PITX3* in our study. Our study expands the scope of the clinical heterogeneity of *PITX3* variants. It also improves our understanding and increases the attention given to patients with *PITX3* variants.

**Supplementary Information:**

The online version contains supplementary material available at 10.1186/s12886-022-02573-x.

## Introduction

Buphthalmos is derived from “ox-eyed” in Greek. It describes the visible enlargement of the eyeball at birth or soon after due to increased intraocular pressure (IOP ) [1]. Primary congenital glaucoma (onset at birth) and primary infantile glaucoma (onset after birth to 3 years) are the most frequent causes of buphthalmos [2, 3]. Corneal oedema, increased corneal diameter, and optic disc cupping are the classical manifestations in patients with buphthalmos [4]. *PITX3* is the third PITX gene in the PITX/RIEG homeobox family and plays a critical role in normal lens development during vertebrate eye formation [5, 6]. *PITX3* is responsible for various ocular defects, including congenital cataract, anterior segment dysgenesis (ASD), Peters’ anomaly, and microphthalmia [7, 8]. In this case, our aim is to report novel phenotype (unilateral buphthalmos and corneal opacity) of a 4-month-old female infant with variants in PITX3.

## Case description

The proband in this study is a 4-month-old female infant. She was born after a full-term uneventful pregnancy and did not suffer a significant perinatal history. Physical examination after birth revealed a birth weight of 3015 g, a head circumference of 34 cm, and a body length of 47 cm. She had no systemic anomalies and no remarkable family history.

**Fig. 1:**
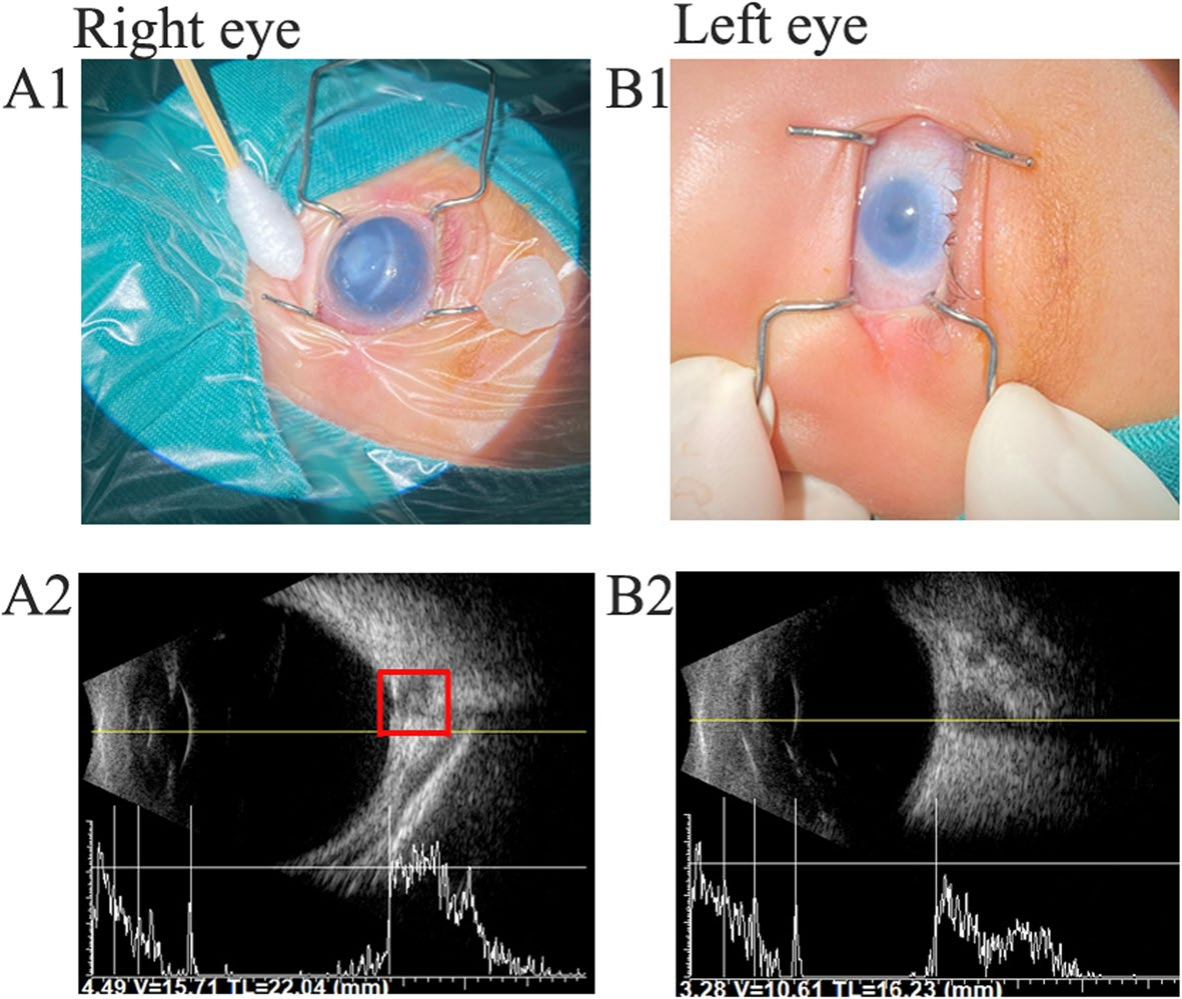
The phenotype of the proband with variant in *PITX3.* Fig. **A1** The photography of the right eye. Buphthalmos with macrocornea (14.50 mm*14.50 mm), corneal opacity and edema were detected. Fig. **A2 **B-scan of the right eye. Buphthalmos with axial length of 22.04 mm was present and an inferiorly decentred excavation within the superficial optic disc tissue revealed in the red box. Fig. **B1 **The photography of the left eye. Microcornea (7.50 mm*6.50 mm) and corneal opacity were detected. Fig. **B2 **Microphthalmia with axial length of 16.23 mm was detected

She was referred to our Eye Clinic because of an enlarged and cloudy right eye. Ophthalmologic examination (including B-scan and slit lamp examination) showed the following manifestations before surgery: right eye buphthalmos with long axial length (22.04 mm), macrocornea with diffuse corneal oedema and opacity (14.50 mm*14.50 mm), left eye microphthalmia (short axial length: 16.23 mm) and microcornea with diffuse corneal oedema and opacity (7.50 mm*6.50 mm) (Fig. 1). The IOP was 23.78 mmHg and 17.30 mmHg in the right and left eyes, respectively. Additionally, an inferiorly decentred excavation within the superficial optic disc tissue was revealed by the B-scan in the right eye (Fig. 1). A 360° trabeculotomy was immediately performed on the right eye. She did not return for routine follow-up. Six months after the trabeculotomy, corneal staphyloma and corneal fistula with iris plugging of the perforated ulcer were detected according to the telephone follow-up. Ophthalmectomy was performed for the right eye at the local hospital.

Informed consent was obtained from the parents of the proband according to the protocol approved by West China Hospital Sichuan University. Whole exome sequencing has been performed on the proband’s genomic DNA sample. S220 Focused-ultrasonicator (Covaris, Massachusetts, USA) was used to shear Genomic DNA (1-3μg) into an average size of 150-bp. The preparation of standard Illumina libraries was conducted by DNA Sample Prep Reagent Set (MyGenostics, Beijing, China).

To acquire the DNA library, genomic DNA (1–3 μg) and the probes were mixed and then PCR amplification was performed. A DNBSEQ-T7RS sequencer for paired reads of 150 bp (average sequencing depth: 1485.68; target area coverage: 10X: 99.93 20X: 99.87) was used for next-generation sequencing. Variants in genes responsible for glaucoma, microphthalmia and macrophthalmia (Table S1) were selected and analysed through multiple bioinformatic analytic steps. Variants with a minor allele frequency (MAF) smaller than 0.01 (based on the 1000 genome, ESP6500, dbSNP, EXAC) and sequencing quality with a coverage of more than 5 were included. Additionally, synonymous variants without a splice site change and benign variants predicted by online tools (SIFT, PolyPhen-2, MutationTaster, GERP++ and REVEL) were excluded.

**Fig. 2:**
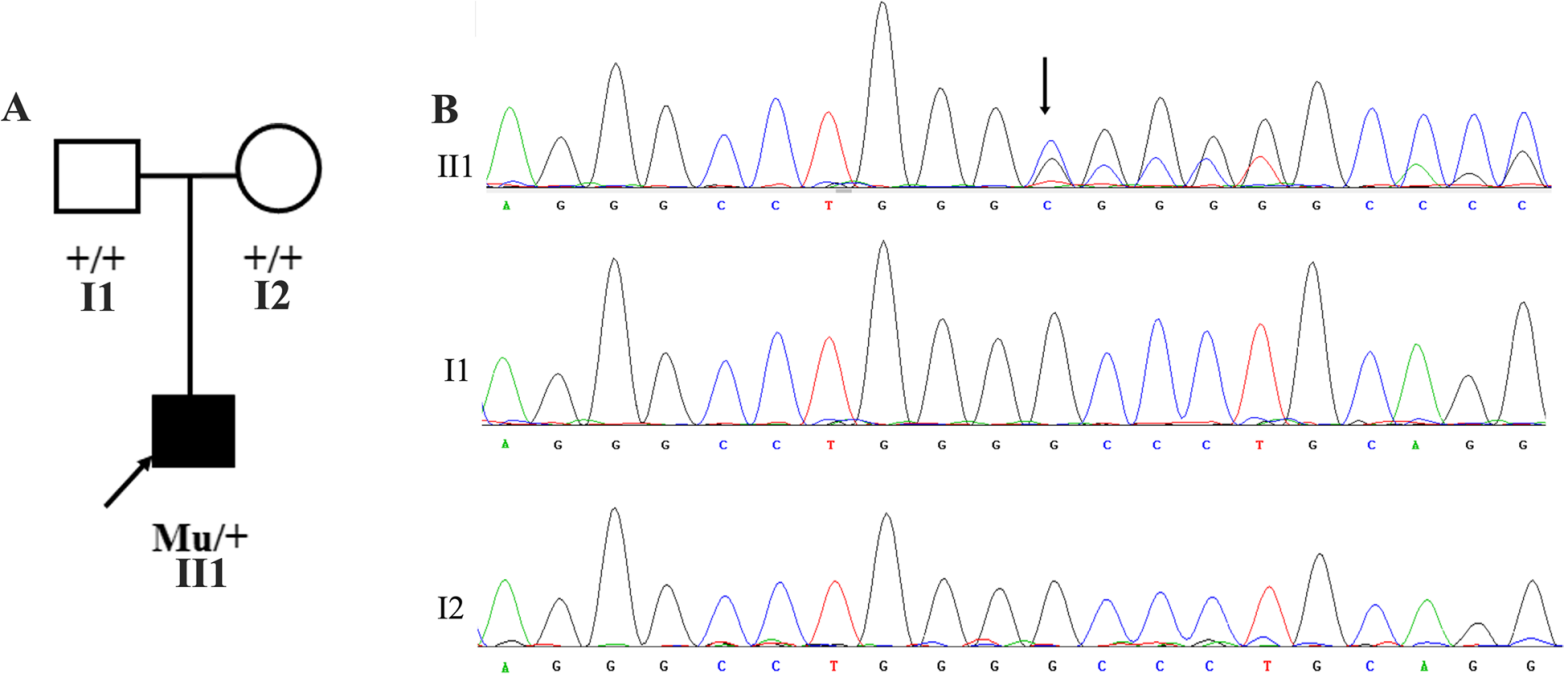
The pedigree and Sanger sequence of the proband and her parents. **A** The pedigree of the family. Squares represent males and circles represent females. An arrow pointing towards the symbol indicates proband individuals. The shaded symbol indicates affected proband. +: wide type, Mu: mutation. **B** the Sanger sequencing of the pathogenic variant in PITX3. The arrow indicates the position of the variant in *PITX3*

Only one truncation in exon 4 (c.640_656dup (p. Gly220Profs*95)) of *PITX3* was identified (Fig. 2). No pathogenic variants were identified in other genes. Sanger sequencing validation, including amplification, sequencing, and target sequence analysis, was performed following a previously described method [9]. Additionally, segregation analysis was conducted, and her healthy parents did not carry the same variant.

## Conclusions

**Fig. 3:**
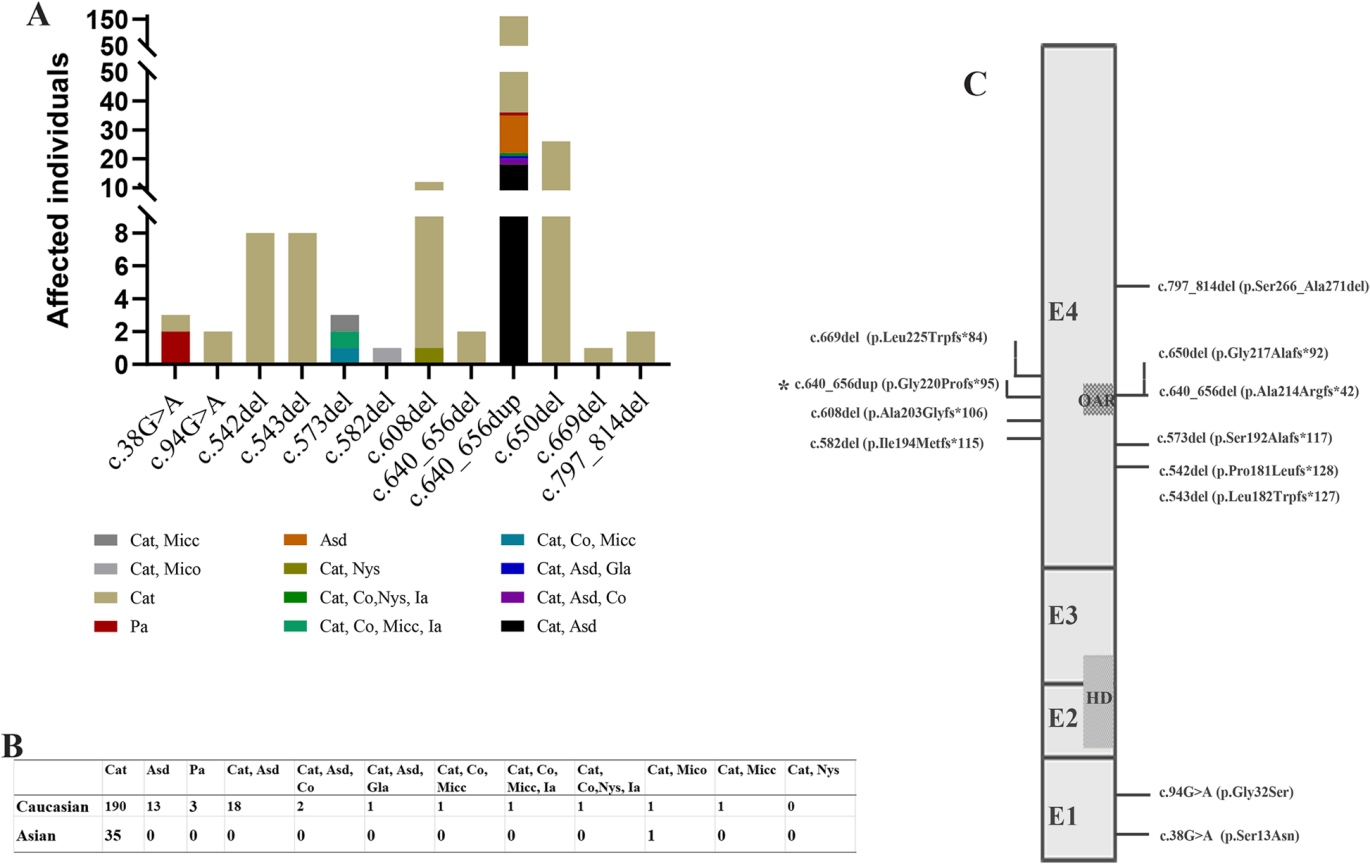
A summary of the phenotype and genotype of *PITX3* with heterozygous variants in previous studies. **A** A summary of the phenotype of the heterozygous individuals in previous studies. Abbreviation: Cat, Cataract; Asd, Anterior segment dysgenesis; Co, Corneal opacity; Pa, Peters anomaly; Micc, Microcornea; Mico, Microphthalmia; Nys, Nystagmus; Ia, iridocorneal adhesions; Gla, Glaucoma. **B** The distribution of the phenotype of Asian and Caucasian with heterozygous variant in *PITX3*. **C** The distribution of the variants in the exons of *PITX3*. Abbreviation: HD: homeo domain; OAR: *Otp/aristaless/rax* domains

PITX3 has been reported to be mapped close to aphakia on mouse chromosome 19. The lens develops normally in mice with *Pitx3* knockdown until an arrest occurs around embryonic Days 10.5–11. This timing corresponds to the moment of initial expression of *Pitx3* in the lens [10]. Microphthalmos or aphakia could be detected in mice with knockdown of *Pitx3* [11]. Mutations of this gene have been reported to be associated with congenital cataract, anterior segment dysgenesis (ASD), Peters’ anomaly, and microphthalmia (Table 1 and Fig. 3).

**Table 1:** Summary of the reported variants in *PITX3*

PMID	Variant	Family	Numbers		Phenotype						
			Family	Family Members	Cataract	Anterior segment dysgenesis	Peters anomaly	Corneal opacity	Microcornea	Microphthalmia	Nystagmus
Homozygous											
21836522	c.640_656del (p.Ala214Argfs*42)	Family 1	1	1	-	-	-	-	-	Y	-
16565358	c.650del (p.Gly217Alafs*92)	Family 1		1	Y	-	-	Y	-	Y	-
16565358	c.650del (p.Gly217Alafs*92)	Family 1		1	Y	-	-	Y	-	Y	-
29405783	c.669del (p.Leu225Trpfs*84)	Family 2	1	2	-	Y	-	-	-	-	-
Heterozygous											
29405783	c.38G>A (p.Ser13Asn)	Family 5	1	1	-	-	Y	-	-	-	-
29405783	c.38G>A (p.Ser13Asn)	Family 5		1	-	-	Y	-	-	-	-
29405783	c.38G>A (p.Ser13Asn)	Family 5		1	Y	-	-	-	-	-	-
9620774	c.94G>A (p.Gly32Ser)	Family 2	1	2	Y	-	-	-	-	-	-
21633712	c.542del (p.Pro181Leufs*128)	Family 1	1	8	Y	-	-	-	-	-	-
26885225	c.543del (p.Leu182Trpfs*127)	Family 1	1	8	Y	-	-	-	-	-	-
24555714	c.573del (p.Ser192Alafs*117)	Family 5	1	1	Y	-	-	Y	Y	-	-
24555714	c.573del (p.Ser192Alafs*117)	Family 5		1	Y	-	-	Y	Y	-	-
24555714	c.573del (p.Ser192Alafs*117)	Family 5		1	Y	-	-	-	Y	-	-
29405783	c.582del (p.Ile194Metfs*115)	Family 4	1	1	Y	-	-	-	-	Y	-
28249924	c.608del (p.Ala203Glyfs*106)	Family 1	1	1	Y	-	-	-	-	-	-
28249924	c.608del (p.Ala203Glyfs*106)	Family 1		1	Y	-	-	-	-	-	Y
28249924	c.608del (p.Ala203Glyfs*106)	Family 1		1	Y	-	-	-	-	-	-
28249924	c.608del (p.Ala203Glyfs*106)	Family 1		1	Y	-	-	-	-	-	-
28249924	c.608del (p.Ala203Glyfs*106)	Family 1		1	Y	-	-	-	-	-	-
28249924	c.608del (p.Ala203Glyfs*106)	Family 1		1	Y	-	-	-	-	-	-
28249924	c.608del (p.Ala203Glyfs*106)	Family 1		1	Y	-	-	-	-	-	-
30816539	c.608del (p.Ala203Glyfs*106)	Family 10003	1	5	Y	-	-	-	-	-	-
9620774	c.640_656dup (p.Gly220Profs*95)	Family 1	1	6	-	Y	-	-	-	-	-
15286169	c.640_656dup (p.Gly220Profs*95)	Family 1	1	6	Y	-	-	-	-	-	-
15286169	c.640_656dup (p.Gly220Profs*95)	Family 1		1	Y	Y	-	-	-	-	-
15286169	c.640_656dup (p.Gly220Profs*95)	Family 2	1	7	-	Y	-	-	-	-	-
15286169	c.640_656dup (p.Gly220Profs*95)	Family 2		4	Y	Y	-	-	-	-	-
15286169	c.640_656dup (p.Gly220Profs*95)	Family 3	1	14	Y	-	-	-	-	-	-
15286169	c.640_656dup (p.Gly220Profs*95)	Family 4	1	12	Y	-	-	-	-	-	-
15286169	c.640_656dup (p.Gly220Profs*95)	Family 5	1	5	Y	-	-	-	-	-	-
15665340	c.640_656dup (p.Gly220Profs*95)	Family 1	1	7	Y	-	-	-	-	-	-
15665340	c.640_656dup (p.Gly220Profs*95)	Family 1		1	Y	Y	-	-	-	-	-
15665340	c.640_656dup (p.Gly220Profs*95)	Family 2	1	6	Y	-	-	-	-	-	-
15665340	c.640_656dup (p.Gly220Profs*95)	Family 2		4	Y	Y	-	-	-	-	-
15665340	c.640_656dup (p.Gly220Profs*95)	Family 3	1	14	Y	-	-	-	-	-	-
16272057	c.640_656dup (p.Gly220Profs*95)	Family 1	1	20	Y	-	-	-	-	-	-
16636655	c.640_656dup (p.Gly220Profs*95)	Family 1	1	29	Y	-	-	-	-	-	-
18989383	c.640_656dup (p.Gly220Profs*95)	Family 1	1	1	Y	-	-	-	-	-	-
18989383	c.640_656dup (p.Gly220Profs*95)	Family 1		1	Y	Y	-	-	-	-	-
18989383	c.640_656dup (p.Gly220Profs*95)	Family 1		1	Y	Y	-	-	-	-	-
18989383	c.640_656dup (p.Gly220Profs*95)	Family 1		1	Y	Y	-	-	-	-	-
18989383	c.640_656dup (p.Gly220Profs*95)	Family 1		1	Y	Y	-	-	-	-	-
18989383	c.640_656dup (p.Gly220Profs*95)	Family 1		1	Y	Y	-	-	-	-	-
18989383	c.640_656dup (p.Gly220Profs*95)	Family 1		1	Y	Y	-	-	-	-	-
18989383	c.640_656dup (p.Gly220Profs*95)	Family 1		1	Y	Y	-	-	-	-	-
18989383	c.640_656dup (p.Gly220Profs*95)	Family 1		1	Y	-	-	-	-	-	-
18989383	c.640_656dup (p.Gly220Profs*95)	Family 1		1	Y	-	-	-	-	-	-
18989383	c.640_656dup (p.Gly220Profs*95)	Family 1		1	Y	-	-	-	-	-	-
18989383	c.640_656dup (p.Gly220Profs*95)	Family 1		1	Y	-	-	-	-	-	-
18989383	c.640_656dup (p.Gly220Profs*95)	Family 1		1	Y	-	-	-	-	-	-
18989383	c.640_656dup (p.Gly220Profs*95)	Family 1		1	Y	-	-	-	-	-	-
18989383	c.640_656dup (p.Gly220Profs*95)	Family 1		1	Y	-	-	-	-	-	-
18989383	c.640_656dup (p.Gly220Profs*95)	Family 1		1	Y	-	-	-	-	-	-
24555714	c.640_656dup (p.Gly220Profs*95)	Family 1	1	1	Y	Y	-	Y	-	-	-
24555714	c.640_656dup (p.Gly220Profs*95)	Family 1		1	Y	Y	-	-	-	-	-
24555714	c.640_656dup (p.Gly220Profs*95)	Family 1		1	Y	-	-	-	-	-	-
24555714	c.640_656dup (p.Gly220Profs*95)	Family 2	1	1	Y	-	-	-	-	-	-
24555714	c.640_656dup (p.Gly220Profs*95)	Family 3	1	1	Y	-	-	-	-	-	-
24555714	c.640_656dup (p.Gly220Profs*95)	Family 3		1	Y	Y	-	Y	-	-	-
24555714	c.640_656dup (p.Gly220Profs*95)	Family 4	1	1	Y	-	-	-	-	-	-
24555714	c.640_656dup (p.Gly220Profs*95)	Family 4		1	Y	-	-	Y	-	-	Y
29405783	c.640_656dup (p.Gly220Profs*95)	Family 1	1	1	Y	Y	-	-	-	-	-
29405783	c.640_656dup (p.Gly220Profs*95)	Family 1		1	Y	-	-	-	-	-	-
29405783	c.640_656dup (p.Gly220Profs*95)	Family 1		1	Y	-	-	-	-	-	-
29405783	c.640_656dup (p.Gly220Profs*95)	Family 3	1	1	-	-	Y	-	-	-	-
29405783	c.640_656dup (p.Gly220Profs*95)	Family 3		1	Y	-	-	-	-	-	-
30816539	c.640_656del (p.Ala214Argfs*42)	Family 10094	1	1	Y	-	-	-	-	-	-
30816539	c.640_656del (p.Ala214Argfs*42)	Family 10178	1	1	Y	-	-	-	-	-	-
16565358	c.650del (p.Gly217Alafs*92)	Family 1	1	26	Y	-	-	-	-	-	-
29405783	c.669del (p.Leu225Trpfs*84)	Family 2		1	Y	-	-	-	-	-	-
30894134	c.797_814del (p.Ser266_Ala271del)	Family 1	1	1	Y	-	-	-	-	-	-
30894134	c.797_814del (p.Ser266_Ala271del)	Family 1		1	Y	-	-	-	-	-	-

PMID	Variant	Family	Phenotype				Country	Ethnicity	Years	Ref.
			Sclerocornea	iridocorneal adhesions	buphthalmos	Glaucoma				
Homozygous										
21836522	c.640_656del (p.Ala214Argfs*42)	Family 1	Y	-	-	-	Saudi Arabia	Caucasian	2011	Aldahmesh et al., 2011 [12]
16565358	c.650del (p.Gly217Alafs*92)	Family 1	-	-	-	-	Lebanese	Caucasian	2006	Bidinost et al 2006 [13]
16565358	c.650del (p.Gly217Alafs*92)	Family 1	-	-	-	-	Lebanese	Caucasian	2006	Bidinost et al 2006 [13]
29405783	c.669del (p.Leu225Trpfs*84)	Family 2	Y	-	Y	-	Iraq	Caucasian	2018	Celia et al,. 2018
Heterozygous										
29405783	c.38G>A (p.Ser13Asn)	Family 5	-	-	-	-	French	Caucasian	2018	Celia et al,. 2018
29405783	c.38G>A (p.Ser13Asn)	Family 5	-	-	-	-	French	Caucasian	2018	Celia et al,. 2018
29405783	c.38G>A (p.Ser13Asn)	Family 5	-	-	-	-	French	Caucasian	2018	Celia et al,. 2018
9620774	c.94G>A (p.Gly32Ser)	Family 2	-	-	-	-	USA	Caucasian	1998	Semina et al., 1998 [14]
21633712	c.542del (p.Pro181Leufs*128)	Family 1	-	-	-	-	UK	Caucasian	2011	Berry et al., 2011 [15]
26885225	c.543del (p.Leu182Trpfs*127)	Family 1	-	-	-	-	Chinese	Asian	2015	Xiangyu Ye et al,.2015
24555714	c.573del (p.Ser192Alafs*117)	Family 5	-	Y	-	-	Belgo-Romanian	Caucasian	2014	Verdin et al., 2014 [6]
24555714	c.573del (p.Ser192Alafs*117)	Family 5	-	-	-	-	Belgo-Romanian	Caucasian	2014	Verdin et al., 2014 [6]
24555714	c.573del (p.Ser192Alafs*117)	Family 5	-	-	-	-	Belgo-Romanian	Caucasian	2014	Verdin et al., 2014 [6]
29405783	c.582del (p.Ile194Metfs*115)	Family 4	-	-	-	-	North Ireland	Caucasian	2018	Celia et al,. 2018
28249924	c.608del (p.Ala203Glyfs*106)	Family 1	-	-	-	-	Chinese	Asian	2017	Liu et al., 2017 [16]
28249924	c.608del (p.Ala203Glyfs*106)	Family 1	-	-	-	-	Chinese	Asian	2017	Liu et al., 2017 [16]
28249924	c.608del (p.Ala203Glyfs*106)	Family 1	-	-	-	-	Chinese	Asian	2017	Liu et al., 2017 [16]
28249924	c.608del (p.Ala203Glyfs*106)	Family 1	-	-	-	-	Chinese	Asian	2017	Liu et al., 2017 [16]
28249924	c.608del (p.Ala203Glyfs*106)	Family 1	-	-	-	-	Chinese	Asian	2017	Liu et al., 2017 [16]
28249924	c.608del (p.Ala203Glyfs*106)	Family 1	-	-	-	-	Chinese	Asian	2017	Liu et al., 2017 [16]
28249924	c.608del (p.Ala203Glyfs*106)	Family 1	-	-	-	-	Chinese	Asian	2017	Liu et al., 2017 [16]
30816539	c.608del (p.Ala203Glyfs*106)	Family 10003	-	-	-	-	Chinese	Asian	2019	Zehua Wu et al,.2019
9620774	c.640_656dup (p.Gly220Profs*95)	Family 1	-	-	-	-	USA	Caucasian	1998	Semina et al., 1998 [14]
15286169	c.640_656dup (p.Gly220Profs*95)	Family 1	-	-	-	-	UK	Caucasian	2004	Berry et al., 2004 [15]
15286169	c.640_656dup (p.Gly220Profs*95)	Family 1	-	-	-	-	UK	Caucasian	2004	Berry et al., 2004 [15]
15286169	c.640_656dup (p.Gly220Profs*95)	Family 2	-	-	-	-	UK	Caucasian	2004	Berry et al., 2004 [15]
15286169	c.640_656dup (p.Gly220Profs*95)	Family 2	-	-	-	-	UK	Caucasian	2004	Berry et al., 2004 [15]
15286169	c.640_656dup (p.Gly220Profs*95)	Family 3	-	-	-	-	English	Caucasian	2004	Berry et al., 2004 [15]
15286169	c.640_656dup (p.Gly220Profs*95)	Family 4	-	-	-	-	Chinese	Asian	2004	Berry et al., 2004 [15]
15286169	c.640_656dup (p.Gly220Profs*95)	Family 5	-	-	-	-	Hispanic	Caucasian	2004	Berry et al., 2004 [15]
15665340	c.640_656dup (p.Gly220Profs*95)	Family 1	-	-	-	-	UK	Caucasian	2005	P K F Addison et al,.2005 [17]
15665340	c.640_656dup (p.Gly220Profs*95)	Family 1	-	-	-	Y	UK	Caucasian	2005	P K F Addison et al,.2005 [17]
15665340	c.640_656dup (p.Gly220Profs*95)	Family 2	-	-	-	-	UK	Caucasian	2005	P K F Addison et al,.2005 [17]
15665340	c.640_656dup (p.Gly220Profs*95)	Family 2	-	-	-	-	UK	Caucasian	2005	P K F Addison et al,.2005 [17]
15665340	c.640_656dup (p.Gly220Profs*95)	Family 3	-	-	-	-	UK	Caucasian	2005	P K F Addison et al,.2005 [17]
16272057	c.640_656dup (p.Gly220Profs*95)	Family 1	-	-	-	-	Brazil	Caucasian	2005	Finzi et al., 2005 [18]
16636655	c.640_656dup (p.Gly220Profs*95)	Family 1	-	-	-	-	Australia	Caucasian	2006	Burdon et al., 2006 [19]
18989383	c.640_656dup (p.Gly220Profs*95)	Family 1	-	-	-	-	Australian	Caucasian	2008	Summers et al., 2008 [20]
18989383	c.640_656dup (p.Gly220Profs*95)	Family 1	-	-	-	-	Australian	Caucasian	2008	Summers et al., 2008 [20]
18989383	c.640_656dup (p.Gly220Profs*95)	Family 1	-	-	-	-	Australian	Caucasian	2008	Summers et al., 2008 [20]
18989383	c.640_656dup (p.Gly220Profs*95)	Family 1	-	-	-	-	Australian	Caucasian	2008	Summers et al., 2008 [20]
18989383	c.640_656dup (p.Gly220Profs*95)	Family 1	-	-	-	-	Australian	Caucasian	2008	Summers et al., 2008 [20]
18989383	c.640_656dup (p.Gly220Profs*95)	Family 1	-	-	-	-	Australian	Caucasian	2008	Summers et al., 2008 [20]
18989383	c.640_656dup (p.Gly220Profs*95)	Family 1	-	-	-	-	Australian	Caucasian	2008	Summers et al., 2008 [20]
18989383	c.640_656dup (p.Gly220Profs*95)	Family 1	-	-	-	-	Australian	Caucasian	2008	Summers et al., 2008 [20]
18989383	c.640_656dup (p.Gly220Profs*95)	Family 1	-	-	-	-	Australian	Caucasian	2008	Summers et al., 2008 [20]
18989383	c.640_656dup (p.Gly220Profs*95)	Family 1	-	-	-	-	Australian	Caucasian	2008	Summers et al., 2008 [20]
18989383	c.640_656dup (p.Gly220Profs*95)	Family 1	-	-	-	-	Australian	Caucasian	2008	Summers et al., 2008 [20]
18989383	c.640_656dup (p.Gly220Profs*95)	Family 1	-	-	-	-	Australian	Caucasian	2008	Summers et al., 2008 [20]
18989383	c.640_656dup (p.Gly220Profs*95)	Family 1	-	-	-	-	Australian	Caucasian	2008	Summers et al., 2008 [20]
18989383	c.640_656dup (p.Gly220Profs*95)	Family 1	-	-	-	-	Australian	Caucasian	2008	Summers et al., 2008 [20]
18989383	c.640_656dup (p.Gly220Profs*95)	Family 1	-	-	-	-	Australian	Caucasian	2008	Summers et al., 2008 [20]
18989383	c.640_656dup (p.Gly220Profs*95)	Family 1	-	-	-	-	Australian	Caucasian	2008	Summers et al., 2008 [20]
24555714	c.640_656dup (p.Gly220Profs*95)	Family 1	-	-	-	-	Belgian	Caucasian	2014	Verdin et al., 2014 [6]
24555714	c.640_656dup (p.Gly220Profs*95)	Family 1	-	-	-	-	Belgian	Caucasian	2014	Verdin et al., 2014 [6]
24555714	c.640_656dup (p.Gly220Profs*95)	Family 1	-	-	-	-	Belgian	Caucasian	2014	Verdin et al., 2014 [6]
24555714	c.640_656dup (p.Gly220Profs*95)	Family 2	-	-	-	-	Belgian	Caucasian	2014	Verdin et al., 2014 [6]
24555714	c.640_656dup (p.Gly220Profs*95)	Family 3	-	-	-	-	Belgian	Caucasian	2014	Verdin et al., 2014 [6]
24555714	c.640_656dup (p.Gly220Profs*95)	Family 3	-	-	-	-	Belgian	Caucasian	2014	Verdin et al., 2014 [6]
24555714	c.640_656dup (p.Gly220Profs*95)	Family 4	-	-	-	-	Belgian	Caucasian	2014	Verdin et al., 2014 [6]
24555714	c.640_656dup (p.Gly220Profs*95)	Family 4	-	Y	-	-	Belgian	Caucasian	2014	Verdin et al., 2014 [6]
29405783	c.640_656dup (p.Gly220Profs*95)	Family 1	-	-	-	-	France	Caucasian	2018	Celia et al,. 2018
29405783	c.640_656dup (p.Gly220Profs*95)	Family 1	-	-	-	-	France	Caucasian	2018	Celia et al,. 2018
29405783	c.640_656dup (p.Gly220Profs*95)	Family 1	-	-	-	-	France	Caucasian	2018	Celia et al,. 2018
29405783	c.640_656dup (p.Gly220Profs*95)	Family 3	-	-	-	-	French	Caucasian	2018	Celia et al,. 2018
29405783	c.640_656dup (p.Gly220Profs*95)	Family 3	-	-	-	-	French	Caucasian	2018	Celia et al,. 2018
30816539	c.640_656del (p.Ala214Argfs*42)	Family 10094	-	-	-	-	Chinese	Asian	2019	Zehua Wu et al,.2019
30816539	c.640_656del (p.Ala214Argfs*42)	Family 10178	-	-	-	-	Chinese	Asian	2019	Zehua Wu et al,.2019
16565358	c.650del (p.Gly217Alafs*92)	Family 1	-	-	-	-	Lebanese	Caucasian	2006	Bidinost et al 2006 [13]
29405783	c.669del (p.Leu225Trpfs*84)	Family 2	-	-	-	-	Iraq	Caucasian	2018	Celia et al,. 2018
30894134	c.797_814del (p.Ser266_Ala271del)	Family 1	-	-	-	-	Chinese	Asian	2019	Fan, Q et al,.2019 [21]
30894134	c.797_814del (p.Ser266_Ala271del)	Family 1	-	-	-	-	Chinese	Asian	2019	Fan, Q et al,.2019 [21]

Notes: *NA* not reported in original article; *F* family; *M* family member; *Het* heterozygous; *Hom* homozygous; *Y* carry the relevant phenotype; −-, normal

Presently, twelve variants have been reported in 32 families. These variants include two missense variants in two families and ten truncations in 32 families [6–8, 12–24]. Four homozygote individuals with more severe phenotypic abnormalities were reported because of consanguineous marriage in three families (Table 1). Six Asian families and 26 Caucasian families have been reported to have these variants in previous studies. Congenital cataracts without other abnormities were more common in Asians than Caucasians with variants in *PITX3*. The c.640_656dup (p.Gly220Profs*95) mutation hot spot was detected in 18 families. For these affected individuals with heterozygous variants, cataracts were the most common manifestations and were detected in 92.74% of patients with *PITX3* variants. Anterior segment dysgenesis and corneal opacity could be found in 14.53 and 2.13% of patients harbouring *PITX3* variants, respectively. Microphthalmia (0.43%), microcornea (1.28%), nystagmus (0.85%), iridocorneal adhesions (0.85%), and glaucoma (0.43%) could also be detected (Table 1 and Fig. 3). However, no studies have reported corneal staphyloma and corneal fistula in patients with *PITX3* variants. Here, we report a 4-month-old female infant carrying a variant in *PITX3.* Unilateral buphthalmos, corneal staphyloma and corneal fistula were detected, and 360° trabeculotomy was conducted on the right eye. However, ophthalmectomy was performed for the right eye at the local hospital because of the protruding opaque cornea and corneal fistula that presented 6 months after the 360° trabeculotomy.

In summary, we report a novel phenotype characterized by unilateral buphthalmos, corneal staphyloma and corneal fistula this is associated with a *PITX3* variant. Our study expands the scope of the clinical heterogeneity of PITX3 variants. It also improves our understanding and increases the attention given to patients with *PITX3* variants.

## Supplementary Information

**Additional file 1.**

## Data Availability

The sequence data were deposited in NCBI Gene bank and can be retrieved using GenBank accession number: BankIt2572599 seq ON236641. Other data and supplementary information are included in this published article.

## Abbreviations

IOP: Increased intraocular pressure
ASD: Anterior segment dysgenesis
ESP: Exome Sequencing Project v. 6500
ExAC: Exome Aggregation Consortium
MAF: Minor allele frequency
dbSNP: The Single Nucleotide Polymorphism database
